# Cell sheet produced from periodontal ligament stem cells activated by PAR1 improves osteogenic differentiation

**DOI:** 10.1590/1807-3107bor-2024.vol38.0079

**Published:** 2024-09-02

**Authors:** Letícia Miquelitto GASPARONI, Tomaz ALVES, Bruno Nunes de FRANÇA, Danilo BALZARINI, Emmanuel ALBUQUERQUE-SOUZA, Ana Clara Fagundes PEDRONI, Emanuel da Silva ROVAI, Aldrin Huamán MENDOZA, Carla Renata SIPERT, Marinella HOLZHAUSEN

**Affiliations:** (a) Universidade Federal de Juiz de Fora – UFJF, School of Dentistry, Department of Dental Clinic, Juiz de Fora, MG, Brazil.; (b) University of North Carolina, Adams School of Dentistry, Division of Comprehensive Oral Health, Chapel Hill, NC, USA.; (c) Universidade São Francisco – USF, School of Dentistry, Bragança Paulista, SP, Brazil.; (d) Universidade de São Paulo – USP, School of Dentistry, Department of Stomatology, São Paulo, SP, Brazil.; (e) Queen Mary University, William Harvey Research Institute, London, UK.; (f) Universidade de São Paulo – USP, School of Dentistry, Department of Restorative Dentistry, São Paulo, SP, Brazil.; (g) Universidade Estadual Paulista – Unesp, Institute of Science and Technology, Division of Periodontics, São José dos Campos, SP, Brazil.

**Keywords:** Cell Culture Techniques, Stem Cells, Receptors, Protease-Activated, Periodontal Ligament, Osteogenesis

## Abstract

Periodontal regeneration is a challenge, and tissue engineering based on periodontal ligament stem cells (PDLSCs) has been shown to be a promising alternative to this process. However, the need for scaffolds has limited the therapeutic use of PDLSCs. In this context, scaffold-free tissue engineering using the cell sheet (CS) technique has been developed as an alternative approach to improve tissue regeneration. Previously, we showed that Protease-activated receptor-1 (PAR1) can regulate PDLSCs. Herein, we evaluate whether PAR1 influences osteogenesis in CSs produced from PDLSCs, without the use of scaffolds. PDLSCs were isolated and immunophenotyped. Then, CSs were obtained by supplementing the culture medium with ascorbic acid (50 µg/mL), and PAR1 was activated through its agonist peptide (100 nM). Scaffold-free 3D CSs were successfully produced from PDLSCs, and they showed higher proliferation potential than isolated PDLSCs. Also, PAR1 activation decreased senescence and improved osteogenic differentiation of CSs by increasing mineralized nodule deposition and alkaline phosphatase concentration; PAR1 also modulated osteogenic markers at the gene and protein levels. We further demonstrated that this effect was regulated by Wnt, TGF-βI, MEK, p38 MAPK, and FGF/VEGF signaling pathways in PDLSCs (p < 0.05%). Overall, PAR1 activation increased osteogenic activity in CSs, emerging as a promising scaffold-free therapeutic approach for periodontal regeneration.

## Introduction

Tooth loss is considered a substantial worldwide public health issue because it negatively affects masticatory functions, esthetics, and quality of life. Periodontitis is a major cause of tooth loss.^1^ After periodontal treatment, repair often occurs through the formation of a long junctional epithelium. However, periodontal regeneration is considered the ideal outcome and consists of the restoration of the lost periodontal tissues leading to the formation of new cementum, alveolar bone, and periodontal ligament (PDL).^2^ Tissue engineering can help in this process as an interdisciplinary approach applied to periodontology in order to restore, maintain, or improve tissue functions by using cells, scaffolds, and bioactive molecules.^3^ On this basis, periodontal ligament stem cells (PDLSCs) have been successfully isolated from human third molars and possess characteristics of mesenchymal stromal cells (MSCs). Because of their multipotent differentiation capacity, PDLSCs are a promising tool for periodontal regeneration.^4^

Stem cell-based therapies using cells seeded onto biodegradable scaffolds or single cell suspension injections have become very promising in recent decades. More recently, cell sheet (CS) technology has also attracted considerable interest. In this method, cells grow with specific stimuli until the cell layer spontaneously detaches from the culture surface without the need for proteolytic enzymes. The advantage of CS engineering is providing a large number of cells and keeping the extracellular matrix (ECM) practically intact, so that no scaffolds are required.^5^

Protease-activated receptor-1 (PAR1), also called thrombin receptor, was firstly described in platelets.^6^ PAR1 expression in the periodontium has been demonstrated in human gingival fibroblasts, gingival epithelial cells, periodontal ligament cells, monocytes, and osteoblasts^7^. Our group previously reported the capacity of PAR1 to improve the osteogenic activity of PDLSCs^8^ and PDL CSs, which highlights this receptor as a promising molecular target for bone tissue engineering. Under a future translational perspective, however, studies that show the role of this receptor under conditions of tissue engineering are still lacking.

The aim of this study is to improve our knowledge about the application of PAR1 in periodontal tissue engineering by evaluating whether PAR1 influences osteogenesis in CSs produced from PDLSCs.

## Methodology

### Cell isolation

Human primary PDLSCs were isolated from completely erupted third molars of healthy subjects (n = 3, two female and one male, aged 22 to 24 years) at the School of Dentistry of the University of Sao Paulo, after approval from the Ethics Committee (No. 2,485,321). After extraction, teeth were transported to the laboratory and rinsed with phosphate buffered saline (PBS, Invitrogen, Carlsbad, USA), and PDL attached to the middle third of the root was gently scaled, cut into small pieces, and cultured for cell growth using explant technique under a humidified 5% CO_2_ atmosphere at 37°C. PDLSCs in passage 4-5 were used for all experiments.

### Immunophenotyping

Immunophenotyping was performed by flow cytometry (LSRFortessa X-20, BD Biosciences, San Jose, USA) through the expression of MSC-related specific surface antigens STRO-1, CD44, CD90, CD146 (positive markers), CD31, CD34 (negative markers) (eBioscience, San Diego, USA), and OCT4, SOX2 (stemness markers) (Abcam, Cambridge, UK), according to the criteria described by the International Society for Cellular Therapy. The results were analyzed using FlowJo™ Software (LLC, Ashland, USA).

### Colony-forming-unit fibroblasts (CFU-Fs)

Cells were seeded onto a 6-well plate at 200 cells/well in triplicate. After 10 days, cells were fixed with 4% paraformaldehyde (Sigma, St Louis, USA) and then stained with 0.1% crystal violet (Certistain, Sigma, St Louis, USA) for 15 min. Images were taken using an inverted optical microscope (Eclipse Ti, Nikon, Japan).

### Cell sheet culture and induction of osteogenic differentiation

CSs were prepared by supplementation with vitamin C (50 µg/mL)^10^. 50,000 cells/cm^2^ were seeded per well in 6-well dishes for 24 h and subsequently incubated with clonogenic (CLO) (α-MEM, 10% FBS, 2 mM L-Glutamine, 100 µg/mL penicillin, 100 µg/mL streptomycin, 0.5 mg/mL amphotericin B, and 50 μg/mL ascorbic acid) or osteogenic (OST) (clonogenic medium + 100 nM dexamethasone, 10 mM β-glycerophosphate) (Sigma, St Louis, USA) induction medium for CS production. The induction medium with stimulation was changed every 3 days up to the 14th day, when CSs began to detach from the well.

### PAR1 activation

In parallel, PDLSCs and CSs were treated with osteogenic medium supplemented with PAR1 agonist peptide (OST + PAR1) (100 nM TFLLR-NH_2_, 1464/1, Tocris Bioscience, UK) ^8^. The induction medium with stimulation was changed every 3 days up to the 14th day.

### Cell proliferation assay

Cells were seeded onto a 96-well plate at 1x10^3^ cells/well in triplicate. The proliferation capacity of PDLSCs was analyzed using the WST-1 Assay Kit (ab65473, Abcam, Cambridge, UK), according to manufacturer’s instructions, and absorbance was measured at 450 nm (Synergy H1 Hybrid Multi-Mode Microplate Reader, BioTek Instruments, Winooski, USA).

### Alizarin Red S staining

Cells were seeded onto a 24-well plate at 50,000 cells/cm^2^ in triplicate. After 2, 7, and 14 days of CS induction, cells were fixed with 4% paraformaldehyde. Then, 2% Alizarin Red S (pH 4.2) (Sigma, St Louis, USA) was incubated with cells under stirring for 30 min. After staining, cells were rinsed with PBS, and images were captured using an inverted optical microscope (Eclipse Ti, Nikon, Japan). To quantify matrix mineralization, stained cells were incubated under stirring in 10% ammonium hydroxide (Sigma, St Louis, MO, USA), and absorbance was measured at 405 nm (Synergy H1 Hybrid Multi-Mode Microplate Reader, BioTek Instruments, Winooski, USA).

### Alkaline phosphatase (ALP) activity

Cells were seeded onto a 24-well plate at 50,000 cells/cm^2^ in triplicate. ALP activity was performed after 2, 7, and 14 days of CS induction using cell lysates as sample (ab83369, Alkaline Phosphatase Assay Kit - Colorimetric, Abcam, Cambridge, UK), according to manufacturer’s instructions.

### qRT-PCR

Cells were seeded onto a 6-well plate at 50,000 cells/cm^2^ in triplicate. RNA was collected after 2, 7, and 14 days of induction. Briefly, total RNA was extracted using TRIzol^®^ reagent (15596018, Invitrogen, Carlsbad, USA), according to the manufacturer’s instructions. RNA concentration and purity (A_260_/A_280_ ratio) was determined using spectrophotometer (Synergy H1 Hybrid Multi-Mode Microplate Reader, BioTek Instruments, Winooski, USA). Isolated total RNA was then subjected to reverse transcription using High-Capacity RNA to cDNA Kit (4387406, Applied Biosystems, Foster City, USA) according to the manufacturer’s instructions. qRT-PCR was performed with TaqMan Universal Master Mix II, with UNG (4440038, Applied Biosystems, Foster City, USA) using the StepOne Plus (Life Technologies, Carlsbad, USA). Each reaction contained 20 ng of the sample. Amplicons were generated using the following probes: Alkaline Phosphatase (ALP, Hs03046558_s1), β-galactosidase (GLB1, Hs01035168_m1), Collagen I (COL1A1, Hs00164004_m1), Osteocalcin (OC, Hs00609452_g1), Osteoprotegerin (OPG, Hs00171068_m1), Osterix (Sp7, Hs01866874_s1), PAR_1_ (Hs00169258_m1), Periostin (POSTN, Hs01566734_m1), Receptor activator of nuclear factor kappa B ligand (RANKL, Hs00243519_m1), Runt-related transcription factor 2 (RUNX2, Hs00231692_m1), β-actin (ACTB, Hs99999903_m1), and GAPDH (Hs02786624_g1) (Applied Biosystems, Foster City, CA, USA). To verify the most stable housekeeping gene between β-actin, and GAPDH, the Normfinder Software (Aarhus University Hospital, Aarhus, Denmark) was used. The stability values were defined by the software and GAPDH was chosen as the best endogenous gene. Thus, the expression levels of the target genes were normalized to GAPDH expression and the relative quantification was determined using the formula 2^-^ΔΔ^CT^.

### Enzyme-linked immunosorbent assay (ELISA)

The supernatant was collected at 2, 7, and 14 days of osteogenic induction in the evaluated groups. The samples were vortexed (30 seg) and then centrifuged (20 min, 1000 rcf, 4°C, Eppendorf Microcentrifuge, 5424R, Hamburg, Germany). COL1 (MBS2506379), OC (MBS2885169), OPG (MBS2508007), PAR1 (MBS733919), POSTN (MBS286029), and RANKL (MBS283899) concentration in the supernatants were determined using a commercial ELISA kit (Mybiosource, San Diego, USA), according to manufacturer’s instructions. Absorbance was measured at 450 nm with correction set to 540 nm (Synergy H1 Hybrid Multi-Mode Microplate Reader, BioTek Instruments, Winooski, USA).

### Inhibition of signaling pathways

To evaluate possible PAR1 signaling pathways during osteogenic differentiation, PDLSCs were grown in clonogenic, osteogenic, or osteogenic + PAR1 medium (OST supplemented with 100 nM PAR_1_ agonist peptide) (controls of inhibition), and inhibition of the Wnt/β-catenin, TGF-βRI, MEK, p38 MAPK, and FGFR/VEGFR pathways was performed in osteogenic and osteogenic + PAR1 medium using 50 ng/mL Wnt/β-catenin inhibitor (Recombinant Human Dkk-1 Protein, 5439-DK, R&D Systems, Minneapolis, USA)^11^, 10 μM TGF-βRI inhibitor (SB431542)^12^, 20 μM MEK inhibitor (PD98059)^13^, 10 μM p38 MAPK inhibitor (SB203580)^14^, 10 μM FGFR/VEGFR inhibitor (SU5402)^14^ (all from Tocris Bioscience, UK), for 2, 7, and 14 days. The induction medium with inhibitors was changed every 3 days up to the 14th day.

### Statistical analysis

Statistical data analysis was performed with Prism 6 (GraphPad Software, San Diego, USA). All analyses were performed considering a significance level of 5%. The statistical tests employed were two-way ANOVA with Tukey’s post-hoc test. Data are presented as mean and standard deviation.

## Results

### Collection, isolation, and immunophenotyping revealed that surface proteins of PDLSCs were characteristic of MSCs

**Figure 1:**
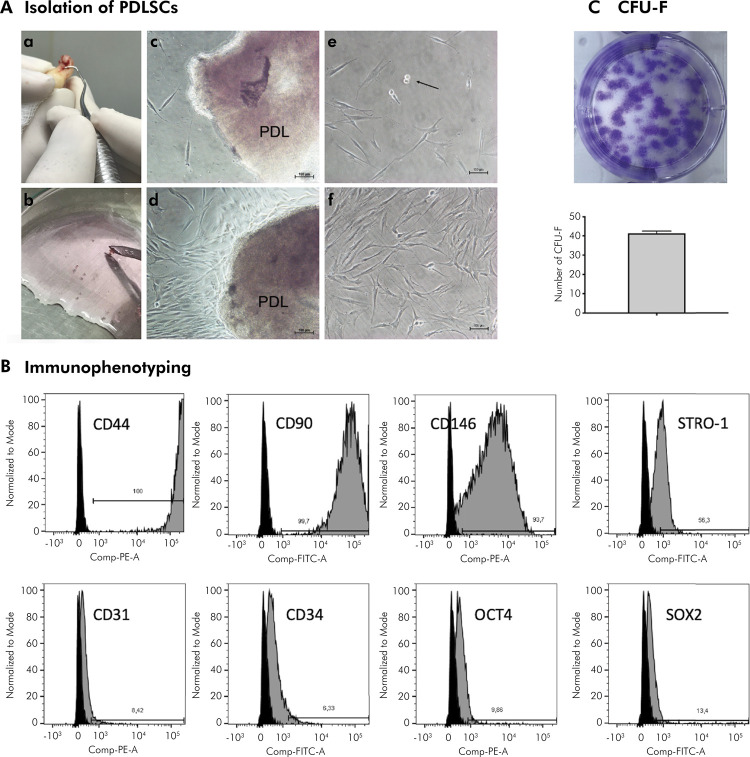
Isolation and characterization of PDLSCs. A) Representative images of a) Macroscopic aspect after scaling the middle third of the root of teeth collected to obtain PDLSCs; b) Fragments of periodontal ligament obtained from root scaling; c) Microscopic aspect of fragments of periodontal ligament obtained during establishment of the primary culture. Cells are observed migrating from the explant after 7 days of cell culture (scale bar = 100 μm); d) Microscopic aspect of the explant after 10 days of cell culture (scale bar = 100 μm); e) Microscopic aspect of PDLSCs after trypsinization, the detail shows cell mitosis (arrow) (scale bar = 100 μm); f) Microscopic aspect of PDLSCs in Passage 0, showing the spindle-like morphologic appearance of isolated PDLSCs (scale bar = 100 μm). B) Immunophenotyping. C) Macroscopic aspect of CFU-F and its quantification.

The PDL collected after root scaling was fragmented and after 7 days in primary culture, PDLSCs were capable to adhere to plastic by migration from the PDL fragment (Figure 1A). PDLSCs formed cells with spindle-like morphology, CFU-Fs (Figure 1B), and showed non-hematopoietic markers similar to a MSC phenotype (Figure 1C).

Analysis of the histograms performed in FlowJo software. Black curve represents the negative control (cells without fluorescent particles).

### Scaffold-free 3D cell sheets can be produced from PDLSCs

**Figure 2:**
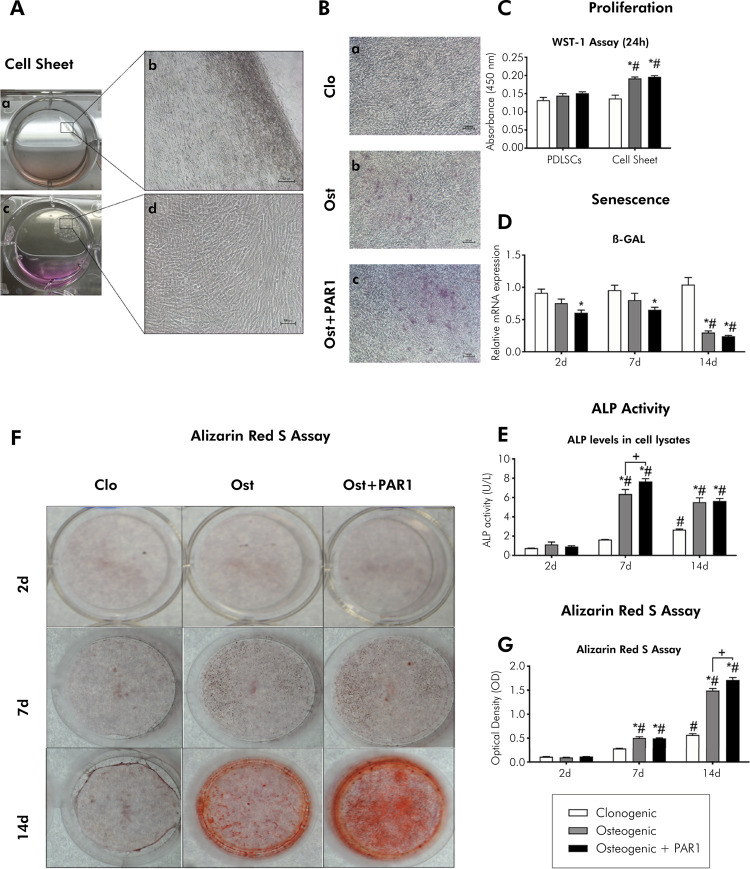
Cell sheet culture and osteogenic differentiation. Representative images. A) a) Macroscopic aspect of cell sheet detaching from the culture plate; b) Microscopic aspect of a detail (scale bar = 100 μm); c) Macroscopic aspect of detached cell sheet; d) Microscopic aspect of a detail (scale bar = 100 μm). B) Microscopic aspect of cultured cell sheets after 14 days of induction of osteogenic differentiation with a) Clonogenic (control), b) Osteogenic, and c) Osteogenic+PAR1 medium (scale bar = 100 μm). C) Cell proliferation (WST-1 Assay). D) Gene expression during induction of osteogenic differentiation in vitro (β-GAL). Calibrating sample: undifferentiated PDLSCs (P4). Housekeeping gene: GAPDH. E) Quantification of alkaline phosphatase (ALP) activity. F) Macroscopic aspect of mineralized nodules formed after 2, 7, and 14 days of induction of osteogenic differentiation by Alizarin Red S staining in the analyzed groups. G) Quantification of Alizarin Red S Assay.

After 14 days in culture under vitamin C stimulation, PDLSCs began to detach from the culture plate without using proteolytic enzymes, being able to form CSs (Figures 2Aa and 2Ab). All experiments were performed at 2, 7 and 14 days before CS detached from the culture plate (Figures 2Ac and 2Ad).

### Cell sheets showed higher proliferation potential and lower senescence than PDLSCs

An increase in the proliferative activity of PDLSCs after CS stimulation (Figure 2B) was observed compared to the non-stimulated control group (p < 0.05%) (Figure 2C). β-galactosidase mRNA levels were downregulated for CSs induced with osteogenic + PAR1 at 2 and 7 days and osteogenic and osteogenic + PAR1 groups at 14 days, showing lower senescence (p < 0.05%) (Figure 2D).

C, D, E and G) White bar indicates clonogenic, gray bar indicates osteogenic, and black bar indicates osteogenic + PAR1 medium group. *Statistically significant difference in relation to the clonogenic group (control) in the same experimental period. ^#^Statistically significant difference in relation to the 2-day experimental period. ^+^Statistically significant difference between groups within the same experimental period. Two-way ANOVA and Tukey’s post-hoc test (p> 0.05). Data are presented as mean and standard deviation.

### PAR1 improves osteogenic differentiation of cell sheets

As expected, an increase in ALP activity at 7 and 14 days was observed for the osteogenic and osteogenic + PAR1 groups, but with a higher increase for the osteogenic + PAR1 group at 7 days (p < 0.05%) (Figure 2E). There was an increase in the formation of mineralized nodules (Figures 2F and 2G) in the CS group treated with osteogenic medium + PAR1-peptide agonist compared to osteogenic medium only, at the 14-day time-point (p < 0.05).

### Osteogenic differentiation potential is different between PDLSCs and cell sheets

**Figure 3:**
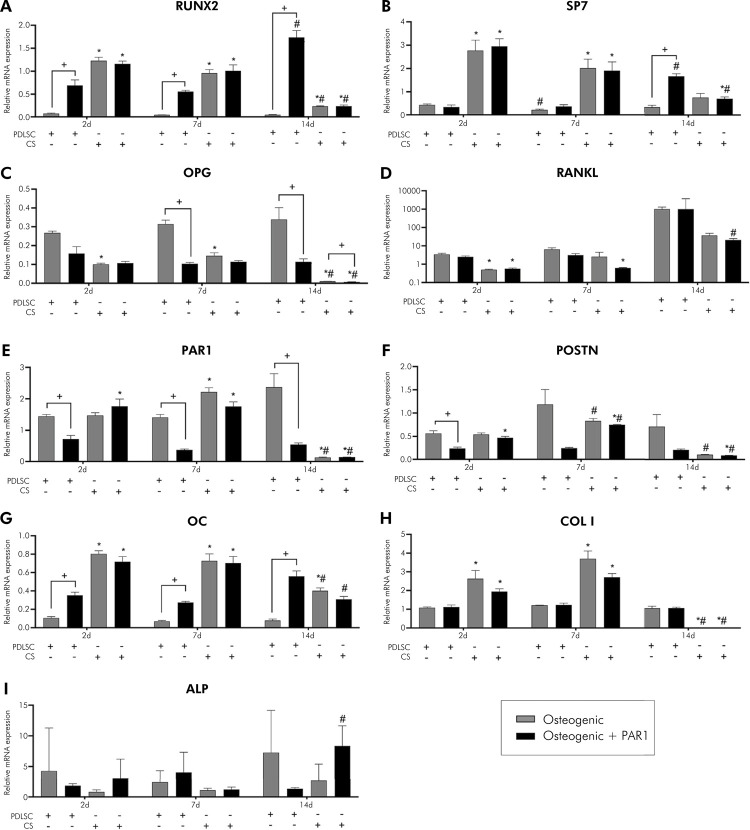
A) to I) Gene expression during induction of osteogenic differentiation in vitro of PDLSCs and cell sheet (RUNX2, SP7, OPG, RANKL, PAR1, POSTN, OC, COL I, and ALP) for 2, 7, and 14 days. Calibrating sample: undifferentiated PDLSCs (P4). Housekeeping gene: GAPDH. Gray bar indicates osteogenic and black bar indicates osteogenic + PAR1 medium group.

**Figure 4:**
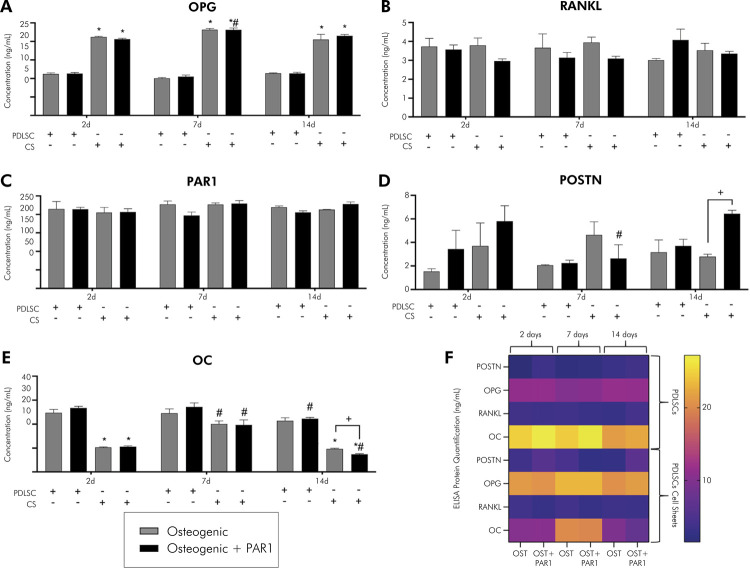
A) to F) Protein levels (ELISA) during induction of osteogenic differentiation in vitro of PDLSCs and cell sheet (OPG, RANKL, PAR1, POSTN, and OC) for 2, 7, and 14 days.

Compared to PDLSCs, CSs exhibited higher gene expression at 2 and 7 days for RUNX2, SP7, PAR1, POSTN, OC, COL I, ALP) (p < 0.05%); on the contrary, OPG and RANKL were downregulated on CSs (Figure 3). In the experimental periods evaluated, there was an increase in gene expression in osteogenic and osteogenic + PAR1 groups in CSs at 7 days (POSTN) and a decrease in gene expression in CSs at 14 days (RUNX2, SP7, OPG, PAR1, POSTN, OC, COL I), compared to the same group at 2 days (p < 0.05%). At the protein level, an elevated synthesis of OPG and a reduction of osteocalcin levels in the supernatant of CSs-treated groups were observed compared to PDLSCs in all experimental time-points (p < 0.05%) (Figures 4A and 4E). There was no difference between CSs and PDLSCs in terms of protein measurement for RANKL, PAR1, and POSTN (Figures 4B–D). In the experimental periods evaluated, there was an increase in levels of the OC protein in osteogenic and osteogenic + PAR1 groups in CSs at 7 days, compared to the same group in experimental period of 2 days (p < 0.05%) (Figure 4F). Overall, these findings showed that CSs conditions improved the osteogenic potential of PDLSCs.

*Statistically significant difference in relation to the equivalent PDLSC group in the same experimental period. ^#^Statistically significant difference in relation to the 2-day experimental period. ^+^Statistically significant difference within the same group in the same experimental period. Two-way ANOVA with Tukey’s post-hoc test (p > 0.05). Data are presented as mean and standard deviation.

Gray bar indicates osteogenic and black bar indicates osteogenic + PAR1 medium group. *Statistically significant difference in relation to the equivalent PDLSC group in the same experimental period. ^#^Statistically significant difference in relation to the 2-day experimental period. ^+^Statistically significant difference within the same group in the same experimental period. Two-way ANOVA with Tukey’s post-hoc test (p> 0.05). Data are presented as mean and standard deviation.

### PAR1 activation influences osteogenic differentiation potential in PDLSCs and cell sheets

**Figure 5:**
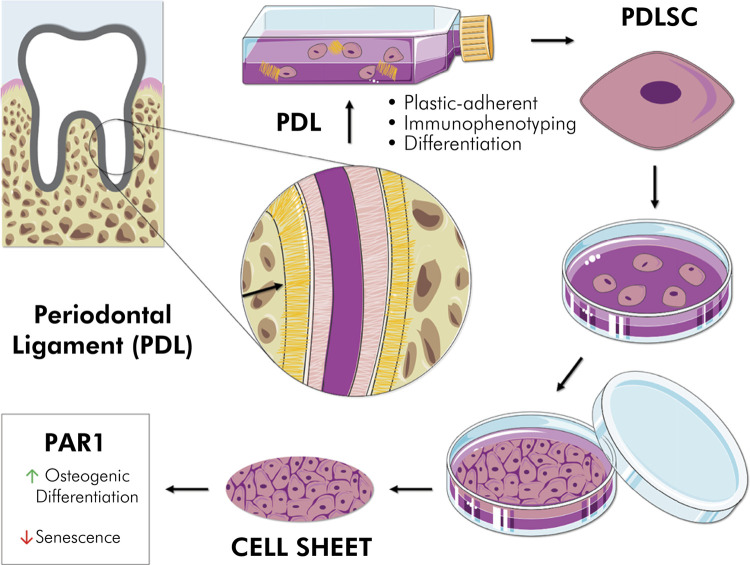
Cell sheet technique. Immediately after extraction, human teeth were transported to the laboratory in a tube with culture medium and antibiotics. The periodontal ligament (PDL) was scaled, fragmented, and cultured in flasks. PDLSCs were isolated and cultured with a specific medium to obtain the cell sheets. PAR1 activation increased osteogenic activity and decreased senescence in cell sheets.

PAR1 activation in PDLSCs led to higher gene expression of RUNX2 and OC at 2, 7, and 14 days and of SP7 at 14 days compared to osteogenic medium. In addition, PAR1 activation in PDLSCs led to lower gene expression of PAR1 at 2, 7, and 14 days, POSTN at 2 days, and OPG at 7 and 14 days, compared to osteogenic medium. In CSs, PAR1 activation decreased gene expression of OPG at 14 days, compared to osteogenic medium. In the experimental periods evaluated, there was an increase in gene expression in the osteogenic + PAR1 group in the PDLSCs at 14 days (RUNX2, SP7) and in the CSs at 14 days (ALP), compared to the same group in experimental period of 2 days (p < 0.05%) (Figure 3). CSs treated with PAR1 agonist showed higher protein levels of POSTN and lower protein levels of OC than CSs treated with osteogenic medium at 14 days (p < 0.05%) (Figures 4D and E). In the experimental periods evaluated, there was an increase in OPG protein levels in osteogenic + PAR1 group in CSs at 7 days, and a decrease in POSTN protein levels in osteogenic + PAR1 group in CSs at 7 days and of OC protein in osteogenic + PAR1 group in PDLSCs and CSs at 14 days, compared to the same group in experimental period of 2 days (p < 0.05%) (Figures 4A–F). To summarize the results of PDLSCs isolation, CSs production, and key outcomes related to PAR1 activation, an illustration was developed (Figure 5).

### Inhibition of Wnt, TGF-βRI, MEK, p38 MAPK, and FGFR/VEGFR signaling pathways influences the profile of genes and proteins related to osteogenesis in PDLSCs

**Figure 6:**
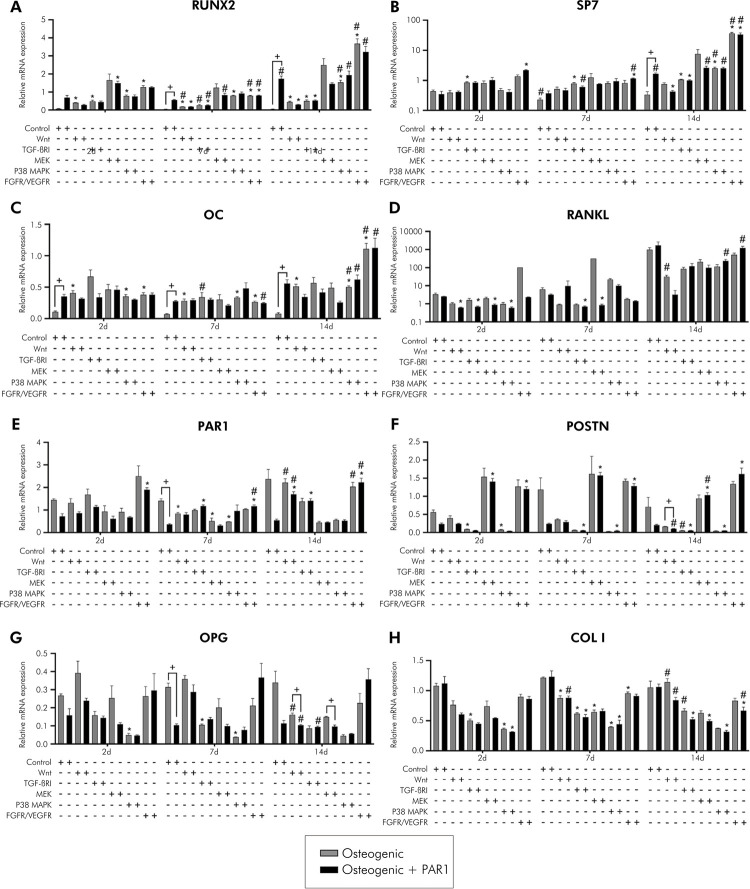
A) to H) Gene expression during induction of osteogenic in vitro differentiation and inhibition of signaling pathways (Wnt/β-catenin, TGF-βRI, MEK, p38 MAPK, and FGFR/VEGFR) of PDLSCs (RUNX2, SP7, OPG, RANKL, PAR1, POSTN, OC, and COL I) for 2, 7, and 14 days. Calibrating sample: undifferentiated PDLSCs (P4). Housekeeping gene: GAPDH.

**Figure 7:**
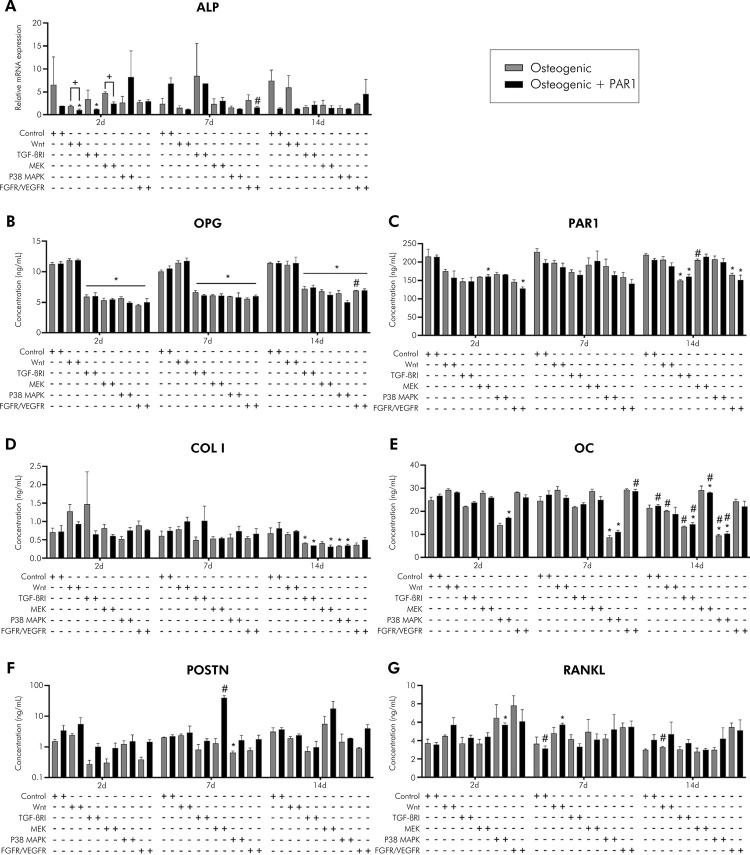
A) Gene expression during induction of in vitro osteogenic differentiation and inhibition of signaling pathways (Wnt/β-catenin, TGF-βRI, MEK, p38 MAPK, and FGFR/VEGFR) of PDLSCs (ALP) for 2, 7, and 14 days. Calibrating sample: undifferentiated PDLSCs (P4). Housekeeping gene: GAPDH. B) to G) Protein levels (ELISA) during induction of in vitro osteogenic differentiation and inhibition of signaling pathways (Wnt/β-catenin, TGF-βRI, MEK, p38 MAPK, and FGFR/VEGFR) of PDLSCs (OPG, RANKL, PAR1, POSTN, OC, and COL I) for 2, 7 and 14 days.

Comparing the osteogenic and osteogenic + PAR1 groups during inhibition of the signaling pathways evaluated with the corresponding control group, RUNX2, SP7 and OC genes were upregulated during inhibition of Wnt, TGF-βRI, MEK, p38 MAPK, and FGFR/VEGFR at 2, 7 and 14 days, as was RANKL at 14 days (Figures 6A–D). PAR1 gene was upregulated during inhibition of Wnt and FGFR/VEGFR at 2 and 14 days and downregulated in the other evaluated groups (Figure 6E). POSTN gene was upregulated during inhibition of MEK and FGFR/VEGFR at 2, 7, and 14 days (Fig. 6F). OPG, COL I, and ALP genes were downregulated in all groups evaluated at 2, 7, and 14 days (Figures 6G-H and 6A), OPG, PAR1, COL I, OC protein levels were lower than control for all groups evaluated at 2, 7 and 14 days (Figures 7B–E). POSTN protein levels were lower than the control at 2 and 7 days and higher than the control during inhibition of MEK at 14 days (Figure 7F). RANKL protein levels were higher than the control during inhibition of Wnt, p38 MAPK, and FGF/VEGFR at 2, 7, and 14 days (Figure 7G) (p<0.05%).

Gray bar indicates osteogenic and black bar indicates osteogenic + PAR1 medium group. *Statistically significant difference in relation to the equivalent control group in the same experimental period. ^#^Statistically significant difference in relation to the 2-days experimental period. ^+^Statistically significant difference within the same group in the same experimental period. Two-way ANOVA with Tukey’s post-hoc test (p > 0.05). Data are presented as mean and standard deviation.

Gray bar indicates osteogenic and black bar indicates osteogenic + PAR1 medium group. *Statistically significant difference in relation to the equivalent control group in the same experimental period. ^#^Statistically significant difference in relation to the 2-day experimental period. ^+^Statistically significant difference within the same group in the same experimental period. Two-way ANOVA with Tukey’s post-hoc test (p> 0.05). Data are presented as mean and standard deviation.

### PAR1 activation influences the inhibition of Wnt, TGF-βRI, MEK, p38 MAPK, and FGFR/VEGFR signaling pathways in PDLSCs

**Figure 8:**
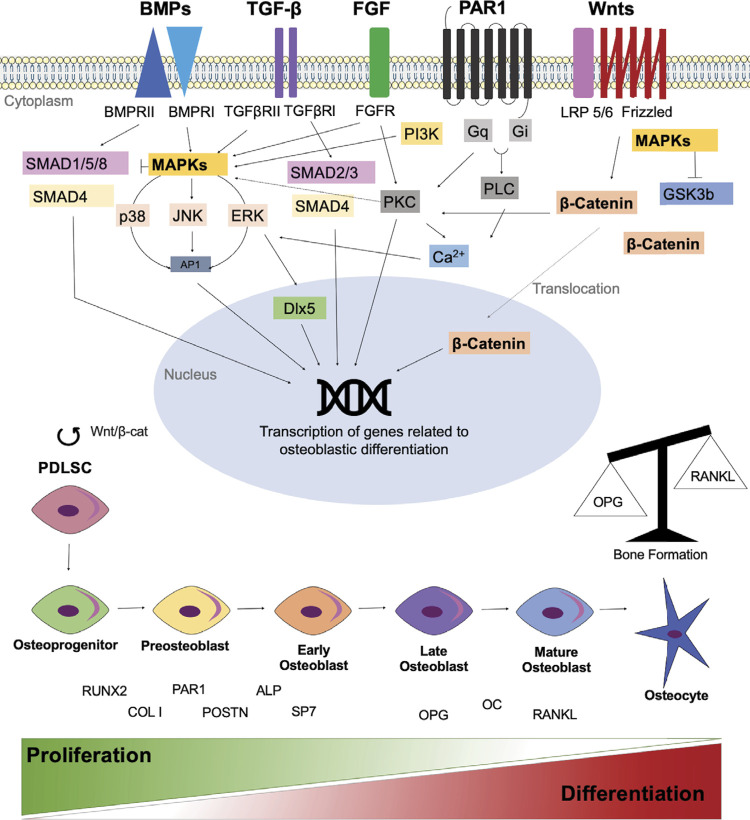
Schematic presentation of signaling pathways involved in osteoblastic differentiation and its connection to the expression of genes and bone-specific proteins.

OPG gene was downregulated for osteogenic + PAR1 compared to osteogenic group during inhibition of Wnt and TGF-βRI at 14 days (Figure 6G), POSTN gene was downregulated during inhibition of Wnt at 14 days (Figure 6F), and ALP gene was downregulated during inhibition of Wnt and MEK at 2 days (Figure 6A). In the experimental periods evaluated, comparing osteogenic + PAR1 to the same group at 2 days, RUNX2 gene was downregulated during inhibition of MEK at 7 days (Figure 6A). SP7 gene was downregulated during inhibition of TGF-βRI and FGFR/VEGFR at 7 days, and during inhibition of MEK at 14 days (Figure 6B). OPG gene was downregulated during inhibition of TGF-βRI at 14 days (Figure 6G). RANKL gene was upregulated during inhibition of p38 MAPK and FGFR/VEGFR at 14 days (Figure 6D). PAR1 gene was downregulated during inhibition of FGFR/VEGFR at 7 days (Figure 6E). POSTN gene was downregulated during inhibition of Wnt and MEK at 14 days (Fig. 6F). OC gene was downregulated during inhibition of FGFR/VEGFR at 7 days (Figure 6C). COL I gene was upregulated during inhibition of Wnt at 7 days and downregulated during inhibition of FGFR/VEGFR at 14 days (Figure 6H). ALP gene was downregulated during inhibition of FGFR/VEGFR at 7 days (Figure 7A) (p < 0.05%). PDLSCs treated with PAR1 agonist showed higher POSTN protein levels during inhibition of MEK at 7 days (Figure 7F), and higher OC protein levels during inhibition of FGFR/VEGFR at 7 days and MEK at 14 days (Figure 7E), comparing osteogenic + PAR1 to the same group at 2 days (p < 0.05%). There was no difference regarding the levels of OPG, RANKL, PAR1, and COL I protein during PAR1 activation for inhibitions of the evaluated signaling pathways (Figure 7B–D, G). For a better understanding of the signaling pathways involved in osteoblastic differentiation and their connection to the expression of specific bone genes, proteins, and PAR activation, an illustration was developed (Figure 8).

## Discussion

A previous study from our group has demonstrated an anabolic role played by PAR1 in the biology of human PDLSCs cultured in a two-dimensional (2D) in vitro environment during osteogenesis^8^. Recently, we also demonstrated that PAR1-activated CSs implanted into subcutaneous tissue of immunosuppressed mice allowed ectopic bone formation^9^. Expanding on this, this study specifically focused on investigating the impact of PAR1 activation on the in vitro osteogenic potential within CSs derived from PDLSCs.

To the best of our knowledge, this work present pioneering evidence that PAR1 activation enhances in vitro osteogenic activity within CSs. This effect was observed through reduced senescence, increased mineralization nodule formation, elevated alkaline phosphatase activity, and influence on the profile of genes and proteins related to osteogenesis. Additionally, the disparities in osteogenic potential between PDLSCs and CSs were explored, shedding light on the role of PAR1 activation in modulating osteogenesis-related signaling pathways. This study investigated how PAR1 influences osteogenesis within CSs, eliminating the need for scaffolds and emerging as a promising avenue for periodontal regeneration.

PDLSCs have been the most promising type of stem cells for periodontal regeneration, as they reside in the perivascular space of the periodontium in the oral cavity. PDLSCs can differentiate into osteoblasts, cementoblasts, periodontal ligaments, peripheral nerves, and blood vessels. Also, these cells can limit immunosuppressive and inflammatory responses by T and B cells.^15^

Currently, different systems have been used in the CS preparation methodology: temperature-responsive,^16^ electro-responsive,^17^ photo-responsive,^18^ pH-responsive,^19^ mechanical,^20^ magnetic^21^ systems, among others. Many improvements to these systems have been done, and these different methodologies can influence the comparison of studies.

The methodology adopted in this study for the production of CSs consists of additional supplementation of the culture medium with ascorbic acid, which has the advantage of being a simpler method for preparing CSs.^5,10^ Ascorbic acid itself participates in ECM synthesis and increases COL I production, which can stimulate the proliferation and differentiation of stem cells in vitro, mimicking the biological environment. CSs obtained with dexamethasone and ascorbic acid have been shown to improve bone formation,^22^ suggesting that the adoption of this induction method instead of other methods could also be beneficial when the target is to form osteogenic-like tissues.

Further, CSs showed higher proliferation potential than PDLSCs, and osteogenic differentiation potential was different between PDLSCs and CSs as well. A recent study showed that the transition from 2D to 3D reorganized the cytoskeleton of MSCs from aligned to multidirectional, increasing the therapeutic potential of stem cells.^23^ One of the main characteristics of the CS technique is the presence of an intact physiological ECM. ECM provides a structural and nutritious microenvironment for cell differentiation and proliferation and this may be the main explanation for the difference observed between PDLSCs and CS produced from PDLSCs.^24^ ECM turnover is regulated by matrix metalloproteinases (MMPs) and it is already known that MMP-1 and -13^25^ and MMP-12^26^ have agonist activity for PAR1. Moreover, treatment with thrombin increased the production of MMP-3 in murine intervertebral discs.^27^ These data suggest that there is a relationship between ECM and PAR1 activation.

Senescence is a cellular response to aging. The increase in senescence can decrease cell proliferation and differentiation, impairing tissue regeneration. Currently, there is no consensus in the literature on promotion or prevention of senescence in CS induction models. Although it has already been reported that CSs can increase spontaneous cell differentiation and progressive senescence,^28^ there are studies showing the opposite, with decreased senescence and activation of telomerase activity.^5^ Here we showed that PAR1 activation decreases senescence in CSs during osteogenic differentiation. β-galactosidase (β-gal) is the most used biomarker for aging and senescent cells, and this study showed that β-gal RNAm levels were downregulated for CSs treated with PAR1 at 2, 7, and 14 days (p < 0.05%), showing that PAR1 activation can decrease senescence during osteogenic differentiation in CSs. A study showed that PAR1 regulates cellular senescence by potentiating macrophage recruitment and subsequently secreting PAR1 agonists that stimulate fibroblasts to produce and activate latent TGF-β leading to fibroblast migration, differentiation, and ECM deposition.^29^

Osteoblast differentiation is regulated by RUNX2, which directly regulates SP7 expression. SP7 can act in the regulation of OC (Osteocalcin, an osteoblast specific gene) and COL I (Type 1 Collagen, important component of ECM). OPG acts as a soluble decoy receptor by binding with RANKL, blocking osteoclastic bone resorption, and therefore enhancing the synthesis of bone matrix. ALP is an early indicator of cellular activity and differentiation.^30^ POSTN is a protein expressed in periosteum and periodontium and plays a vital role in regulating bone metabolism.^31^ In addition, this study showed that in most of osteogenesis-related genes studied (RUNX2, SP7, PAR1, OC, and COL I), mRNA levels were upregulated for CSs compared to the same concentrations in PDLSCs. The same was observed for protein levels of OPG. It is also worth mentioning that PAR1 increased POSTN protein levels in CSs at 14 days. These genes and proteins are involved in different stages of osteogenesis regulation. RUNX2 and COL I are early markers, related to the proliferative phase. PAR1, POSTN, ALP, and SP7 are early/intermediate markers, related to matrix maturation phase, while OPG, OC and RANKL are late markers, related to mineralization/remodeling phase. These data are consistent with the results obtained during the increase of mineralization nodule formation (Alizarin Red S) and ALP activity in the PAR1 group compared to the control.

The Wnt pathway represents one of the main mechanisms for development of mesenchymal precursors in osteoprogenitor cells in the early stage of bone formation.^32^ TGF-β1 and FGF are known to maintain progenitor cells and mediate their growth and differentiation.^33,34^ MEK is a kinase enzyme that phosphorylates MAPK, ERK, p38, and, JNK.^35^ PAR1 potentiates Wnt activation of the β-catenin pathway but blocks the JNK pathway.^36^ It has been demonstrated that the PAR1-induced proliferation in astrocytes via MAPK involves multiple signaling pathways, and activation of PARs stimulates ERK1/2 phosphorylation.^37^ In addition, multiple endothelial G protein-coupled receptors agonists stimulate p38 activation.^38^ Thrombin cleave high molecular weight FGF-2, and this can be involved in a variety of physiopathological settings.^39^ Crosstalk between FGF and thrombin signaling pathways, both of which play important roles in tissue repair and angiogenesis, has been reported, as thrombin induces the release of FGF1 from null PAR1 fibroblasts.^40^

This study showed that PAR1 activation influences the profile of genes and proteins related to osteogenesis. The experimental periods analyzed in this work are more related to the early and intermediate stages than to the late stages of bone formation. Even so, it was possible to observe that the blocking of Wnt and TGF-βRI signaling pathways was more significant in the early stage of osteoblast differentiation, while the blocking of the MEK, p38 MAPK, and FGFR/VEGFR signaling pathways were more significant for intermediate/late stages of osteoblast differentiation. PAR1 activation acted on Wnt and MEK signaling pathway in the three stages of osteoblastic differentiation evaluated (Figures 4C, F and 5A). However, this mechanism is not yet fully understood and further studies could elucidate it.

## Conclusion

In summary, scaffold-free 3D CSs can be successfully produced from PDLSCs. Furthermore, this study reveals that PDLSCs and CSs show different proliferation and differentiation potentials. PAR1 activation decreases senescence in CSs during osteogenic differentiation. There was also a difference in the profile of genes and proteins during the inhibition of signaling pathways, and PAR1 activation increased osteogenic activity in CSs, which shows promise as a therapeutic approach for periodontal regeneration.
